# Global mRNA Degradation during Lytic Gammaherpesvirus Infection Contributes to Establishment of Viral Latency

**DOI:** 10.1371/journal.ppat.1002150

**Published:** 2011-07-21

**Authors:** Justin M. Richner, Karen Clyde, Andrea C. Pezda, Benson Yee Hin Cheng, Tina Wang, G. Renuka Kumar, Sergio Covarrubias, Laurent Coscoy, Britt Glaunsinger

**Affiliations:** 1 Department of Plant and Microbial Biology, University of California Berkeley, Berkeley, California, United States of America; 2 Department of Molecular and Cell Biology, University of California Berkeley, Berkeley, California, United States of America; 3 Division of Infectious Diseases and Immunity, School of Public Health, University of California Berkeley, Berkeley, California, United States of America; Emory University, United States of America

## Abstract

During a lytic gammaherpesvirus infection, host gene expression is severely restricted by the global degradation and altered 3′ end processing of mRNA. This host shutoff phenotype is orchestrated by the viral SOX protein, yet its functional significance to the viral lifecycle has not been elucidated, in part due to the multifunctional nature of SOX. Using an unbiased mutagenesis screen of the murine gammaherpesvirus 68 (MHV68) SOX homolog, we isolated a single amino acid point mutant that is selectively defective in host shutoff activity. Incorporation of this mutation into MHV68 yielded a virus with significantly reduced capacity for mRNA turnover. Unexpectedly, the MHV68 mutant showed little defect during the acute replication phase in the mouse lung. Instead, the virus exhibited attenuation at later stages of *in vivo* infections suggestive of defects in both trafficking and latency establishment. Specifically, mice intranasally infected with the host shutoff mutant accumulated to lower levels at 10 days post infection in the lymph nodes, failed to develop splenomegaly, and exhibited reduced viral DNA levels and a lower frequency of latently infected splenocytes. Decreased latency establishment was also observed upon infection via the intraperitoneal route. These results highlight for the first time the importance of global mRNA degradation during a gammaherpesvirus infection and link an exclusively lytic phenomenon with downstream latency establishment.

## Author Summary

Herpesviruses are ubiquitous pathogens infecting a wide range of hosts. They exhibit a biphasic lifecycle comprised of a lytic replicative stage and a relatively dormant latent stage. Members of the gammaherpesvirus subfamily include the human pathogens Epstein-Barr virus and Kaposi's sarcoma-associated herpesvirus, the etiologic agents of some of the most common AIDS-associated malignancies. During a lytic gammaherpesvirus infection, cellular gene expression is severely compromised in a process termed host shutoff. In this study, we address the purpose of this activity in the viral lifecycle through the generation of a single amino acid point mutant selectively defective for host shutoff activity. Unexpectedly, host shutoff appears largely dispensable for viral replication, yet plays an important role in the ability of the virus to traffic and establish latency in the host organism. These findings link two distinct phases of the viral lifecycle and indicate that the global manipulation of gene expression may contribute to the establishment of a lifelong gammaherpesvirus infection.

## Introduction

A number of viruses from diverse evolutionary lineages down-regulate cellular gene expression through a variety of mechanisms [1], [2], [3], [4]. For certain viruses this host shutoff activity is a consequence of viral gene expression strategies, such as influenzavirus cap snatching [5] or picornavirus translation factor cleavage to facilitate alternate mechanisms of ribosome engagement [6]. For others, the contribution of host shutoff towards viral replication and gene expression is not as apparent, although proposed roles include resource reallocation and immune evasion [6], [7]. In many cases, the precise *in vivo* function has been difficult to delineate, in part due to the multifunctional nature of the viral proteins driving host shutoff and the lack of appropriate model systems. Within this latter category are the gammaherpesviruses, which direct host shutoff primarily through the induction of host mRNA degradation.

Gammaherpesviruses include the human pathogens Epstein-Barr virus (EBV/HHV-4) and Kaposi's sarcoma-associated herpesvirus (KSHV/HHV-8) [8], the etiologic agents of some of the most common AIDS-associated cancers, such as Burkitt's lymphoma, nasopharyngeal carcinoma, and primary effusion lymphoma. The related murine gammaherpesvirus 68 (MHV68) is often used as a model for human gammaherpesviruses because, unlike KSHV and EBV, it is genetically tractable, readily replicates in tissue culture without the use of chemicals or overexpression to overcome latency, and infects small rodents including laboratory mice [9], [10], [11]. MHV68 has therefore been instrumental in the identification of factors that contribute to *in vivo* replication and pathogenesis of the gammaherpesviruses.

All herpesviruses exhibit a biphasic lifecycle. In the latent state few viral genes are expressed, and the viral genome is maintained in the nucleus of the host cell until stimulating signals induce lytic reactivation [12], [13], [14]. Most gammaherpesvirus-induced diseases are associated with latency. During the lytic cycle the vast majority of viral genes are expressed, and the virus undergoes active replication to produce progeny virions. At this stage during productive replication, gammaherpesviruses induce global mRNA degradation via the product of the alkaline exonuclease gene, termed SOX in KSHV, muSOX (ORF37) in MHV68, and BGLF5 in EBV [1], [15], [16]. SOX and its homologs are conserved in all subfamilies of herpesviruses, where they were first identified as DNA exonucleases critical for the resolution of branched structures that arise during viral DNA replication [17], [18], [19], [20]. Only in members of the gammaherpesvirus subfamily does the SOX protein have an additional mRNA turnover function. Although the precise mechanism by which these proteins cause mRNA degradation remains unclear, biochemical evidence suggests that at least EBV BGLF5 and KSHV SOX possess some intrinsic ribonuclease (RNase) activity *in vitro* [21], [22]. In addition to mRNA turnover, SOX promotes the nuclear relocalization of cytoplasmic poly(A) binding protein (PABPC), which leads to aberrant polyadenylation and nuclear retention of mRNAs [23], [24]. The failure to repopulate the cytoplasm with newly transcribed mRNAs is thought to increase the overall magnitude of the host gene expression blockade [23].

The contribution of host shutoff towards the gammaherpesviral lifecycle is unknown, and efforts to delete muSOX from MHV68 yielded virus unable to replicate, presumably due to an essential role for the DNase activity [16]. Previous studies found the DNase and host shutoff functions of SOX and BGLF5 to be genetically separable [25], [26], making it possible to evaluate the role of each function of the protein in isolation. We report herein the identification of a functionally similar MHV68 muSOX point mutant that renders the virus selectively defective for host shutoff, enabling the analysis of the role of this phenotype during infection of cultured cells and *in vivo*. The mutant virus exhibited little to no defect during multi-step growth curves in tissue culture or during acute replication in the mouse lung following intranasal inoculation. Unexpectedly, the virus accumulated to significantly reduced levels in the lymph nodes at 10 days post infection and was highly attenuated at the downstream stage of latency establishment. Once trafficking was bypassed via an intraperitoneal infection, the host shutoff defective virus still was attenuated in establishing latency in the spleen. Our results identify for the first time a key role for the lytic mRNA turnover activity in establishing viral latency, emphasizing the important interplay between these seemingly disparate stages of the viral lifecycle.

## Results

### Identification of a Host Shutoff Defective muSOX Mutant

We sought to evaluate the contribution of muSOX-induced mRNA turnover towards the gammaherpesvirus lifecycle through the generation of a muSOX mutant lacking mRNA turnover activity but retaining the conserved DNase function. We engineered mutations in the sites necessary for host shutoff activity in muSOX homologs from other gammaherpesviruses (EBV BGLF5 [25] and KSHV SOX [26]), as well as in several sites selectively conserved amongst these homologs that lay outside the putative DNase domains [27]. However, none of the mutants generated had the desired single-function phenotype (Table S1).

**Figure 1:**
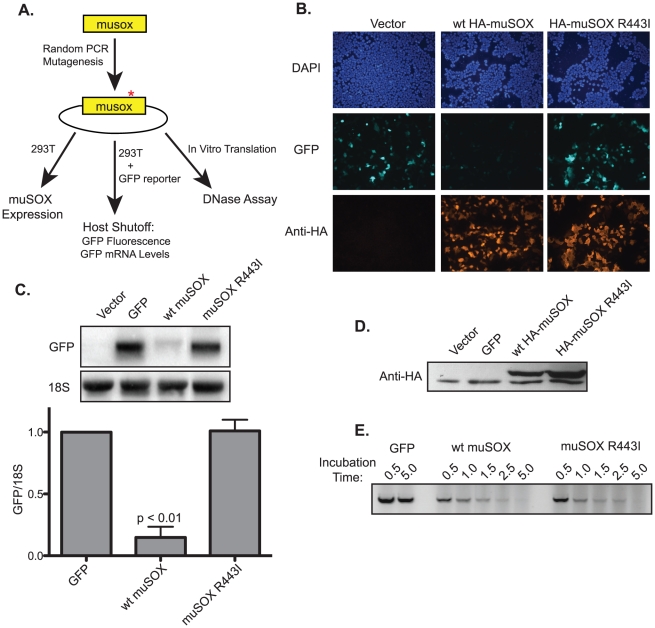
Isolation of the single-function muSOX mutant R443I. (A) Diagram of the random PCR mutagenesis screening strategy. (B) HEK293T cells were transfected with a plasmid expressing GFP alone or together with a plasmid expressing HA-tagged wild-type (wt) muSOX or muSOX R443I. GFP fluorescence was monitored 24 h post transfection, along with HA-muSOX, which was detected by immunofluorescence with anti-HA antibodies. Samples were co-stained with DAPI to visualize nuclei. (C) Cells were transfected for 24 h as described above, whereupon GFP mRNA and 18S rRNA levels were analyzed by Northern blot. Shown is a representative northern blot for GFP mRNA and 18S rRNA, and below is the normalized data from five independent experiments with means and standard deviations shown. (D) Total protein was harvested 24 h post transfection with the indicated plasmids, and Western blotted with anti-HA antibodies. The bottom band present in all samples is due to non-specific binding of the antibody. (E) *In vitro* translated GFP, wild-type muSOX, or muSOX-R443I was incubated with linear DNA for the indicated times. DNA was then extracted and separated by agarose gel electrophoresis. A representative figure from three independent experiments is shown.

We therefore designed an unbiased screen to investigate a large number of mutants following the previous methodology used to identify the single-function SOX variants (Figure 1A) [26]. Briefly, the muSOX gene was amplified by error-prone PCR under conditions designed to introduce 1–4 mutations per gene. The PCR product was then cloned into a mammalian expression vector, and clones were screened for the ability to repress expression of a GFP reporter in a fluorescence-based assay in 293T cells. Mutants that did not deplete GFP fluorescence were categorized as host shutoff defective and further analyzed for wild-type protein expression levels by Western blotting, as even minor changes to the muSOX primary amino acid sequence often significantly reduced its protein expression. Finally, candidate mutants were tested for retention of DNase activity using an *in vitro* assay established previously for SOX and BGLF5 [25], [26]. After screening approximately 150 candidates, we identified a clone that lacked mRNA degradation activity of the GFP reporter, but retained wild-type protein expression levels and DNase activity. Sequencing revealed this clone to have only a single non-silent mutation, the amino acid substitution R443M, which we modified to R443I for purposes of screening by restriction digest. MuSOX R443I failed to suppress GFP protein expression in the fluorescent-based assay for host shutoff (Figure 1B), which was a consequence of its inability to degrade GFP mRNA (Figure 1C). R443I is expressed at slightly higher levels than wild-type (WT) muSOX (Figure 1D), as expected of a mutant that cannot promote turnover of its own mRNA. Importantly, WT and R443I muSOX both degrade linear DNA with very similar kinetics (Figure 1E). We conclude that the amino acid substitution R443I produces a single-function muSOX mutant selectively defective for host shutoff activity.

### Generation and *in vitro* Characterization of MHV68.ΔHS

**Figure 2:**
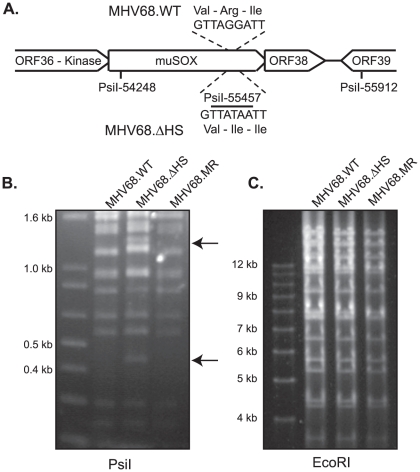
Generation of the MHV68.ΔHS virus. (A) Outline of the strategy for generating MHV68-muSOX-R443I (MHV68.ΔHS), in which the muSOX amino acid arginine at position 443 was mutated to isoleucine, creating an additional PsiI site. The number is derived from the sequenced MHV68 genome [10] (Refseq: NC_001826). (B) MHV68.WT, MHV68.ΔHS, and MHV68.MR BAC DNA were digested with the enzyme PsiI and subsequently resolved on an agarose gel to confirm successful generation of the desired mutants. Arrows indicate the new 1200 bp and 450 bp bands generated after introduction of the PsiI restriction site in muSOX R443I. (C) MHV68.WT, MHV68.ΔHS, and MHV68.MR BAC DNA were digested with the enzyme EcoRI and subsequently resolved on an agarose gel to confirm no unexpected recombination had occurred.

To investigate the role of muSOX-induced host shutoff during viral infection, we engineered a host shutoff defective MHV68 virus by replacing WT muSOX with muSOX R443I using bacterial artificial chromosome (BAC)-based homologous recombination [28]. The R443I mutation introduced an additional PsiI restriction site within the muSOX gene, allowing us to screen for successful recombination via restriction digest (Figure 2A). We also generated a mutant rescue (MR) virus by replacing the R443I mutant with WT muSOX to ensure that any observed phenotypes could be attributed to the R443I mutation rather than to unexpected secondary mutations elsewhere in the viral genome. Restriction digests with PsiI and EcoRI confirmed that both recombinant viruses were successfully isolated and did not have any unexpected recombination events (Figures 2B & 2C). These viruses shall henceforth be referred to as MHV68.ΔHS (host shutoff defective virus) and MHV68.MR (mutant rescue virus). Sequencing of muSOX and the surrounding genomic regions in both recombinants confirmed that only the desired changes were introduced.

**Figure 3:**
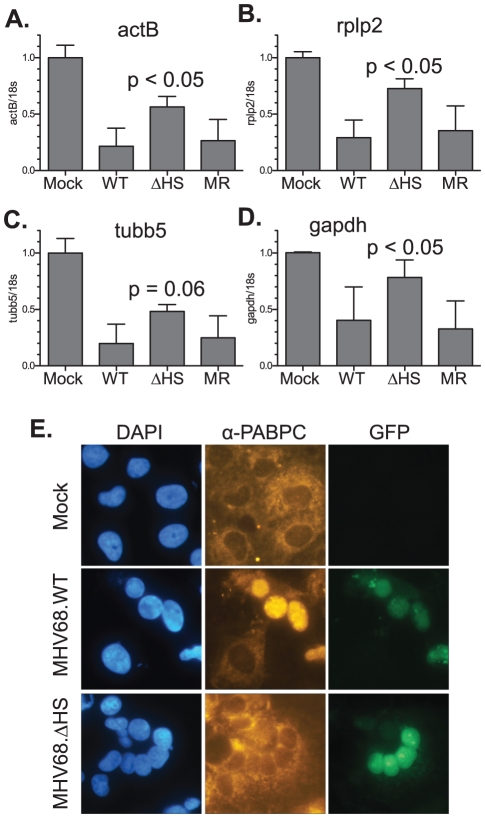
MHV68.ΔHS is defective for host shutoff. RNA was isolated from NIH 3T3 cells infected with MHV68.WT, MHV68.ΔHS, or MHV68.MR at an MOI of 10 at 20 h post infection. ActB (A), Rplp2 (B), Tubb5 (C), GAPDH (D) and 18S RNA levels were quantified via RT-qPCR. The mean and standard deviation of the normalized mRNA/18S ratio is plotted for at least four independent experiments. The p-values comparing MHV68.ΔHS and MHV68.MR are indicated. (E) COS7 cells were infected with MHV68.WT or MHV68.ΔHS for 24 h. Both viruses express GFP from the BAC vector sequence, which serves as a marker for infection. PABPC localization was monitored by immunofluorescence with anti-PABPC antibodies, and cells were stained with DAPI to visualize nuclei.

We next verified that the R443I mutation in MHV68.ΔHS conferred a host shutoff defect during viral infection. We assayed levels of several endogenous mRNAs by real-time quantitative PCR (RT-qPCR) following infection of NIH 3T3 fibroblasts with wild-type MHV68 (MHV68.WT), MHV68.ΔHS, or MHV68.MR. WT and MR infections reduced endogenous β-actin (actB), tubulin-β (tubb5), rplp2, and gapdh mRNA levels to 20–40% of the mock infected sample. In contrast, mRNA levels remained significantly higher in MHV68.ΔHS infected cells (Figure 3A–3D). Thus, the single amino acid change R443I in muSOX is sufficient to suppress virus-induced endogenous mRNA turnover. The somewhat lower endogenous mRNA levels in MHV68.ΔHS compared to mock infected cells could be due either to incomplete inactivation of the muSOX host shutoff function, or to the contribution of one or more additional viral genes towards host shutoff.

An additional phenotype linked to host shutoff caused by muSOX and its homologs is the relocalization of cytoplasmic poly(A) binding protein (PABPC) into the nuclei of infected cells [24]. We observed clear nuclear relocalization of PABPC in cells infected with MHV68.WT, whereas in mock infected or cells infected with MHV68.ΔHS, PABPC remained cytoplasmic (Figure 3E). Thus, MHV68.ΔHS is defective for two hallmarks of host shutoff, mRNA depletion and PABPC relocalization. These results also demonstrate for the first time that muSOX-induced host shutoff is necessary to drive PABPC relocalization during a viral infection.

**Figure 4:**
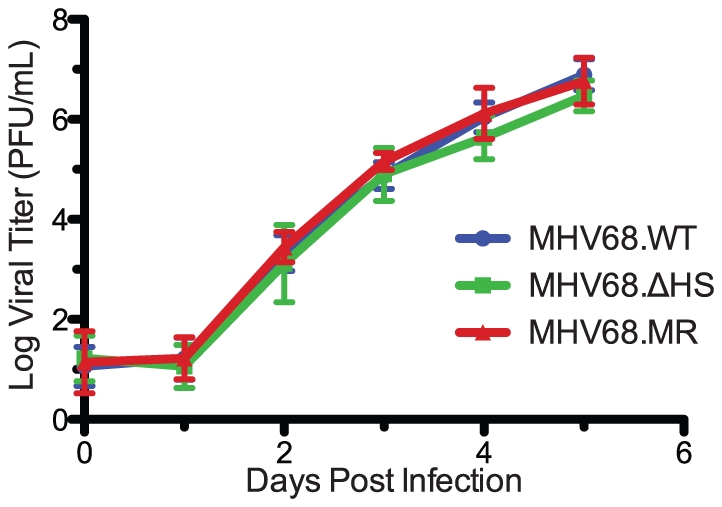
MHV68.ΔHS replicates with wild-type kinetics. Viral replication kinetics were determined by multi-step growth curves in mouse fibroblasts cells following an infection at a MOI of 0.1 with MHV68.WT, MHV68.ΔHS, or MHV68.MR. At the indicated times post infection virus was harvested, and the titer was determined by plaque assay. The mean and standard deviation from at least three independent experiments is graphed.

Previous studies with MHV68 mutants have shown that muSOX is critical for replication in cultured cells [16], [19]. To determine the contribution of host shutoff activity towards this defect, we performed a multi-step growth curve in murine fibroblasts. 3T3 cells were infected at a multiplicity of infection (MOI) of 0.1 plaque forming units (pfu) per cell and MHV68.WT, MHV68.ΔHS, and MHV68.MR all replicated to equivalent titers and with similar kinetics (Figure 4). We sequenced the muSOX gene from MHV68.ΔHS samples at 5 days post-infection (dpi) and found no evidence for reversion or secondary mutations (data not shown). Therefore, muSOX-dependent mRNA degradation had little effect on viral titers in 3T3 cells through multiple replication cycles.

### MHV68.ΔHS Causes Larger Plaques to Develop and Increased Expression of Lytic Cycle Proteins

**Figure 5:**
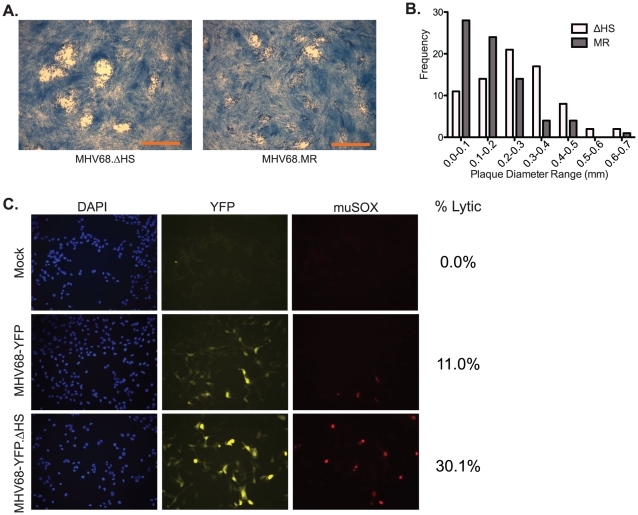
MHV68.ΔHS generates larger plaques and increases the percentage of lytic antigen-expressing cells. (A) Shown are representative images of plaques generated 4 dpi in 3T3 cells infected with MHV68.ΔHS or MHV68.MR. The scale bar in the lower right corner of the images represents 1 mm. (B) Plaque diameters of 75 plaques from 5 independent experiments were measured at 4 dpi from cells infected with MHV68.ΔHS or MHV68.MR, and the frequency distribution was graphed. (C) 3T3 cells infected at an MOI of 1 with either MHV68-YFP or MHV68-YFP.ΔHS were analyzed at 18 hpi for YFP and muSOX expression by immunofluorescence using anti-muSOX antibodies. Samples were co-stained with DAPI to visualize nuclei. Shown are representative images from four independent experiments. The numbers of lytically infected cells (YFP- and muSOX-positive) were determined by counting multiple fields of view from four independent experiments, and the percentage of infected cells expressing muSOX is shown.

Interestingly, we noticed that plaques derived from MHV68.ΔHS infections differed morphologically from those obtained upon MHV68.WT or MHV68.MR infections (Figure 5A). To quantify these differences, we measured 75 plaques from 5 independent MHV68.ΔHS and MHV68.MR infections and found that, indeed, MHV68.ΔHS plaques were generally larger (p-value <0.01) and had a broader frequency distribution (Figure 5B). This seemed unlikely to be a consequence of more rapid lytic replication, given the above growth curve results. In addition, the altered plaque size does not appear to be caused by enhanced cell-to-cell spread of the mutant virus, as we observed no significant differences in the ratio of extracellular to intracellular virus produced in 3T3 cells infected with MHV68.WT, MHV68.ΔHS, and MHV68.MR (data not shown).

We and others have consistently observed that plaques formed upon infection with wild-type (or mutant rescue) MHV68 are often quite heterogeneous in size. One possible explanation for this is asynchronous entry into the lytic cycle, in which smaller plaques arise from later entry into the lytic cycle. In this regard, we sought to determine whether the large plaque phenotype of MHV68.ΔHS might be due to enhanced entry into the lytic cycle, signified by an increased percentage of infected cells expressing early lytic proteins relative to the wild-type virus. To test this hypothesis, we acquired a version of MHV68 (MHV68-YFP) which constitutively expresses YFP from a CMV promoter, regardless of whether infected cells are in the lytic or latent phase [29]. Thus, YFP expression serves as a general marker of infection, whereas lytically infected cells are identified by immunofluorescence-based detection of the early lytic protein muSOX. NIH 3T3 cells were infected at an MOI of 1 with either MHV68-YFP or a version of MHV68-YFP bearing the muSOX R443I mutation (MHV68-YFP.ΔHS), and the percentage of cells co-expressing muSOX and YFP was calculated at 18 hours post infection. A similar number of YFP-expressing cells were detected in cultures infected with MHV68-YFP and MHV68-YFP.ΔHS, indicating equivalent efficiency of initial infection (Figure 5C & data not shown). However, there was an approximately three-fold increase in the percent of cells co-expressing both YFP and muSOX following infection with MHV68-YFP.ΔHS compared to MHV68-YFP (30.1% versus 11.0%; Figure 5C). A similar trend was observed when the YFP- and muSOX-expressing cell populations were analyzed by flow cytometry following infection with these viruses (Figure S1). Also in agreement with these findings were increased levels of the viral ORF54 transcript following a MHV68.ΔHS infection relative to MHV68.WT or MHV68.MR (Figure S2). Thus, enhanced and/or more rapid expression of lytic genes occurs in cells infected with MHV68.ΔHS, perhaps escalating entry into the lytic cycle and leading to larger plaques.

We also evaluated whether increased cell death might contribute to the larger plaques observed following a MHV68.ΔHS infection. We first measured necrosis by quantifying levels of lactate dehydrogenase released into the media at 16 hpi, but detected no measurable release following infection with any of the viruses (Figure S3A). We next monitored apoptosis by several assays, including measuring caspase activity, and testing for loss of plasma membrane symmetry and increased membrane permeability. While there was a slight, but statistically significant, increase in caspase 3/7 activity following MHV68.ΔHS infection relative to MHV68.WT or MHV68.MR infection, in all three cases the level was below that observed in mock infected cells (Figure S3B), making it unlikely that this is a primary cause of the increased plaque size. Plasma membrane asymmetry was monitored using PE-conjugated Annexin V, which binds to a component of the plasma membrane normally found on the cytoplasmic surface, and membrane permeability was quantified by the nucleotide binding dye 7-Amino-Actinomycin (7-AAD). By flow cytometry we detected no significant increase in Annexin V binding or 7-AAD staining following infection with either MHV68.ΔHS or MHV8.MR relative to the uninfected control (Figure S3C). Collectively, these findings indicate that although a higher percentage of MHV68.ΔHS infected cells express viral lytic antigens, this does not appear to cause enhanced cell death, nor does it culminate in higher viral titers.

### Host Shutoff is Dispensable for Acute Replication *in vivo*

The presumed natural route of MHV68 infection is through the upper respiratory tract [30], whereupon the virus undergoes lytic replication in the mucosal epithelial cells lining the mouse lung and nasal cavity. Following intranasal inoculation, viral loads in the lung peak 5–8 dpi before subsiding as the virus traffics to the draining lymph nodes and ultimately to the spleen [31]. A burst of lytic replication occurs in these sites of latency establishment [30], [32] which seeds subsequent latency, primarily in germinal center B cells, although also in macrophages and dendritic cells [33]. By 14–18 dpi splenomegaly develops, and as many as one out of 100 splenocytes harbor the latent viral genome [34].

**Figure 6:**
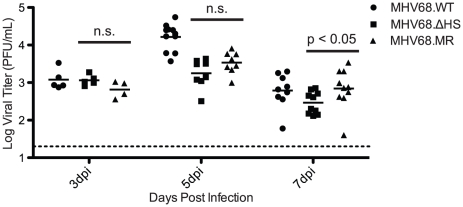
MHV68.ΔHS replicates to near MHV68.MR levels during the acute phase of infection in the mouse lung. C57BL/6 mice were infected intranasally with 5×10^4^ pfu MHV68.WT, MHV68.ΔHS, or MHV68.MR. At 3, 5, or 7 dpi lungs were harvested and homogenized, and viral titers were determined by plaque assay. Each point on the graph represents the viral titer from a single lung, and the bar indicates the mean titer for each virus. The dotted line represents the limit of detection at 20 pfu/lung. The p-values comparing MHV68.ΔHS and MHV68.MR are indicated. “n.s.” indicates a p-value greater than 0.05.

Host shutoff manifests with delayed early kinetics during lytic replication, consistent with the onset of muSOX expression [16]. We therefore hypothesized that MHV68.ΔHS would be attenuated during lytic viral replication in the lung. We infected C57BL/6 mice intranasally with 5×10^4^ pfu of MHV68.WT, MHV68.ΔHS, or MHV68.MR, and monitored viral titers in the lung at 3, 5, and 7 dpi by plaque assay. Viral titers were equivalent at 3 dpi for all three viruses, and at 5 and 7 dpi we observed only a very modest 2–3 fold defect in MHV68.ΔHS relative to MHV68.MR accumulation (Figure 6). Although MHV68.WT reached higher titers than MHV68.MR at 5 dpi, the two viruses accumulated to equivalent levels at all earlier and later timepoints (see subsequent sections). To ensure MHV68.ΔHS had not reverted back to WT or incorporated any compensatory mutations, we sequenced the muSOX gene from infected lung homogenate but found no changes. Thus, contrary to our hypothesis, host shutoff appears largely dispensable for acute replication of gammaherpesviruses *in vivo*.

### MHV68.ΔHS is Attenuated in Latency Establishment

**Figure 7:**
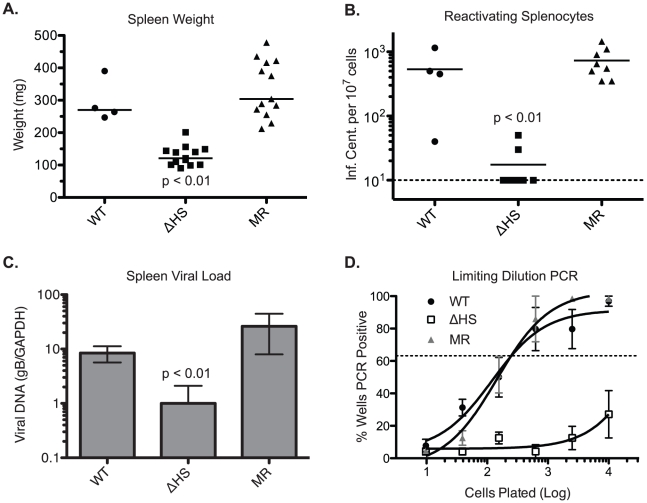
MHV68.ΔHS is attenuated for latency establishment. In two independent experiments, C57BL/6 mice were infected intranasally with 5×10^4^ pfu MHV68.WT, MHV68.ΔHS, or MHV68.MR, and spleens were harvested at 17 dpi. (A) The weight of each spleen is plotted with the mean weight for each virus variant indicated by the bar. (B) Spleen cells were harvested and analyzed for lytic reactivation frequency via infectious center assay. The number of reactivating splenocytes per 10^7^ cells is plotted with the mean for each virus indicated by the bar. The dashed line denotes the limit of detection. (C) DNA was isolated from spleen cells and analyzed by qPCR for the viral glycoprotein B (gB, ORF8) gene and endogenous GAPDH. The normalized gB/GAPDH ratio is plotted along with the mean and standard deviation for each viral infection. The p-values comparing MHV68.ΔHS to MHV68.MR are indicated. (D) The frequency of latently infected cells was determined by limiting dilution-PCR. The percent of wells positive for PCR product is plotted for each dilution. Each point represents the average and standard deviation of three or four mice. The dotted line at 63.2% is used to calculate the frequency of genome harboring cells according to the Poisson distribution.

To evaluate the role of host shutoff during later stages of the viral lifecycle, mice were infected intranasally with 5×10^4^ pfu of MHV68.WT, MHV68.ΔHS, or MHV68.MR, and the spleens were harvested at 17 dpi, during the peak of latency establishment. Interestingly, mice infected with MHV68.ΔHS did not display the characteristic splenomegaly and had spleens three to four times smaller than MHV68.WT and MHV68.MR infected mice (Figure 7A), indicating a role for host shutoff in viral pathogenesis.

To evaluate the cause of MHV68.ΔHS attenuation, we first performed an infectious center assay to test whether the splenocytes from infected mice harbored latent virus capable of lytic reactivation. In this assay, equivalent numbers of splenocytes from infected mice are overlayed onto cultured mouse fibroblasts. Spontaneous lytic reactivation of the splenocytes leads to infection of the fibroblasts, which can be quantified by counting the resulting plaques [34]. Splenocytes from MHV68.ΔHS infected mice reactivated at a significantly lower frequency than those from MHV68.WT or MHV68.MR infections (Figure 7B). This reactivation frequency was independently assessed in a subset of the samples by a limiting dilution cytopathic effect assay, which yielded identical results (data not shown). Plaque assays on spleen homogenates confirmed that no preformed virus was present, indicating that the virus detected in these experiments originated from latently infected cells.

The reduction in lytic reactivation frequency could either be a consequence of a decreased number of latently infected splenocytes or, alternatively, a failure of latently infected cells to undergo lytic reactivation. To distinguish these possibilities, we first monitored viral DNA load in the spleens of infected mice using a previously described qPCR assay for the glycoprotein B (gB) gene [35] and found that MHV68.ΔHS infected splenocytes harbor fewer viral genomes than splenocytes infected with MHV68.WT or MHV68.MR (Figure 7C). We also analyzed the frequency of splenocytes harboring the viral genome by limiting dilution PCR. Briefly, serial dilutions of splenocytes were plated in a 96 well plate and subjected to nested PCR for the viral ORF50 gene [36]. Spiked template controls confirmed that in this assay we were achieving near single copy sensitivity for the viral genome, with a very low rate of false positives (data not shown). While approximately one out of 200 splenocytes latently harbor the MHV68.WT or MHV68.MR genome, the frequency of MHV68.ΔHS genome harboring splenocytes was less than one out of 10,000 splenocytes (Figure 7D). Thus, while host shutoff appears dispensable for acute replication in the lung, it plays an important role in the downstream events leading to efficient latency establishment in the spleen.

### MHV68.ΔHS Exhibits Defects in Both Trafficking and Latency Establishment

**Figure 8:**
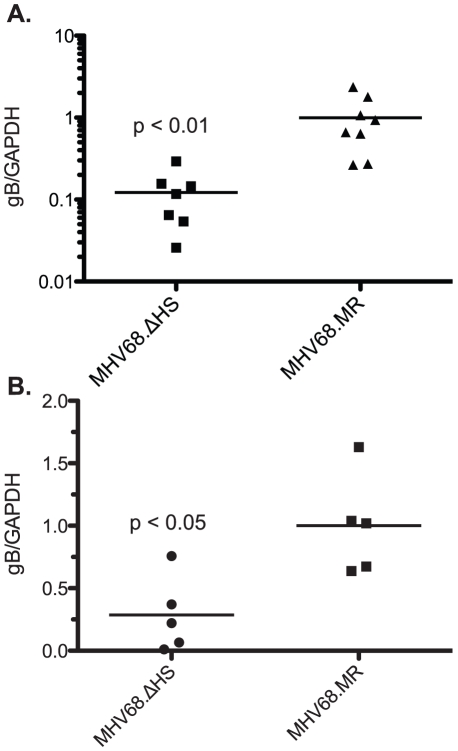
MHV68.ΔHS fails to traffic to the lymph system and establish latency. (A) C57BL/6 mice were infected intranasally with 5×10^4^ pfu MHV68.WT, MHV68.ΔHS, or MHV68.MR, and cervical lymph nodes were harvested at 10 dpi. DNA was isolated from the cells and analyzed by qPCR for the viral glycoprotein B (gB, ORF8) gene and endogenous GAPDH. The normalized gB/GAPDH ratio is plotted along with the mean for each viral infection. (B) Mice were infected intraperitoneally with 1×10^3^ pfu of each of the indicated viruses. DNA was isolated and quantified as above and the normalized gB/GAPDH ratio is plotted along with the mean for each viral infection. Each point on the graph represents the ratio from a single mouse. Data for each figure are compilations of two independent experiments and the p-values are indicated.

During the normal course of an infection, the virus traffics from the site of lytic replication in the lung to the draining lymph nodes and spleen where latency is established. We reasoned that the decreased frequency of latently infected splenocytes during MHV68.ΔHS infection could be a consequence of a defect in trafficking, persistence at these sites, or a latency establishment defect. We quantified levels of virus in the cervical lymph node at 10 dpi, an intermediate time point after peak virus replication in the lung but before peak latency establishment in the spleen. Following an intranasal inoculation, high levels of MHV68 have been found in the cervical lymph node at this time point [30], [32]. Interestingly, we detected significantly lower levels of MHV68.ΔHS at this site relative to MHV68.MR (Figure 8A), suggesting that muSOX-induced mRNA degradation contributes to the ability of the virus to traffic to or persist at the sites of latency establishment.

To determine whether this was the root cause of the defect in latency establishment upon MHV68.ΔHS infection, we next infected mice via the intraperitoneal route, which bypasses the requirement for the virus to undergo acute replication and traffic through the lymph before reaching the spleen [37]. At 19 dpi, we found significantly less MHV68.ΔHS virus in the spleen relative to MHV68.MR by qPCR (Figure 8B), indicating that a defect in viral trafficking cannot fully account for the latency establishment defect observed during an intranasal infection. Collectively, these data indicate that muSOX-induced host shutoff plays important roles both in the ability of MHV68 to traffic to the lymph nodes, as well as establish latency in the spleen.

## Discussion

All gammaherpesviruses studied to date block host gene expression through widespread mRNA degradation, yet the contribution of this function towards the viral lifecycle remained unknown. Here we analyzed the impact of host shutoff both in tissue culture and *in vivo* infections with the murine gammaherpesvirus MHV68, and we found this activity to be largely dispensable for acute lytic replication yet critical for the downstream viral accumulation in the lymph nodes and subsequent establishment of latency in the spleen. These results were unexpected given that MHV68-induced host shutoff is executed by muSOX, a viral protein whose expression has only been detected during the lytic cycle [38], [39], [40]. In addition, the role for muSOX-induced mRNA turnover clearly diverges from that of the alphaherpesvirus host shutoff factor vhs, which similarly induces global mRNA degradation but plays a critical role during acute replication *in vivo* [41], [42], [43]. The severe attenuation of HSV-1 and HSV-2 vhs mutants is hypothesized to be a consequence of ineffective immune evasion, as vhs blocks dendritic cell activation and down-regulates the type I interferon response [44], [45], [46]. In contrast, we find little difference between MHV68.MR and MHV68.ΔHS during acute replication in mice, suggesting that while alpha- and gammaherpesviruses block host gene expression via analogous mechanisms, the functional ramifications of this activity are distinct, and may relate to the unique *in vivo* biology of each class of virus.

Modeling the muSOX three-dimensional structure from the KSHV SOX or EBV BGLF5 crystal structure [21], [47] indicates that amino acid R443 is positioned on the outer surface, away from the catalytic core where nucleic acids are presumably cleaved (data not shown). Although SOX, BGLF5, and muSOX are functionally homologous and likely induce mRNA degradation through a similar mechanism, the identified amino acid mutations that selectively remove host shutoff activity are not conserved, nor are they positioned in the same three-dimensional region. Likely these different mutations alter the local structure of each protein thereby inhibiting or weakening the mRNA or co-factor interactions which contribute to overall mRNA degradation. In this regard, we anticipate that the residual host shutoff activity observed upon infection with the MHV68.ΔHS virus is at least partially due to incomplete inactivation of muSOX mRNA turnover activity by the R443I mutation.

Host shutoff has been hypothesized to play a role in the diversion of gene expression resources and machinery towards the virus during lytic replication [16], [48], as well as general immune evasion through the down-regulation of immune stimulatory factors [49]. Our failure to detect a significant replication defect for MHV68.ΔHS during a multi-step growth curve in cultured murine fibroblasts or in the mouse lung implies that resource reallocation is unlikely to be the primary role for muSOX-induced mRNA depletion. Likewise, the similar viral titers in the lungs of mice infected with MHV68.MR or MHV68.ΔHS argues against a role for muSOX in the general suppression of host innate immune responses Because MHV68.ΔHS still retains residual mRNA degradation activity, it is possible that some degree of host shutoff could be important for viral replication. However, the appearance of significant defects in downstream viral events even with a partial host shutoff defect further strengthens our conclusion that host shutoff plays a vital role in the lifecycle and pathogenesis of MHV68 *in vivo*.

The first major defect observed in an *in vivo* MHV68.ΔHS infection is significantly lower levels of the virus in the lymph nodes at 10 dpi. Following an intranasal infection, wild-type MHV68 undergoes lytic replication in the lung and upper respiratory track, after which the virus drains to the lymph nodes and spleen where a variety of cell types are infected, including macrophages, dendritic cells, and B-cells [37], [50], [51], [52]. A burst of lytic replication occurs at these sites [30], [32], after which replicating virus is cleared and long-term latency is established. Lower levels of MHV68.ΔHS in the lymph node could be caused by a cell-type specific role for muSOX-dependent mRNA degradation in viral replication or immune evasion during viral transport to, and maintenance in, the lymphatic tissue. The observation that the EBV muSOX homolog (BGLF5) down-regulates HLA class I molecules and CD8^+^ T cell recognition in cultured cells may support this model [25]. The means by which the virus traffics to the sites of latency establishment remain unclear, yet it is likely that latently infected B cells carry the virus given that viremia is undetectable during an infection, and latently infected B cells are present in the lung very early after infection [37], [53], [54]. A failure of MHV68.ΔHS to establish latency in these cells might also cause their selective immune-based eradication and decreased accumulation in the lymph nodes. Alternatively, muSOX could influence the ability of the virus to reactivate from latency, as this process has been linked to efficient latency establishment. Mutations in a number of genes alter the frequency of lytic reactivation and lead to lower levels of latency establishment following an intranasal infection, such as v-cyclin (ORF72) [55], M1 [56], M2 [57], [58], and LANA (ORF73) [59], [60], but unlike muSOX these genes are all expressed during latency as well as during lytic replication [61]. Mutations in the lytic ORF36 gene encoding a protein kinase also lead to defects in latency establishment and reactivation [62], [63], which have been tracked to the requirement for ORF36 to inhibit the IRF-3 mediated type I interferon response [62] and its modification of the DNA-damage response protein H2AX [63]. Experiments are currently underway to examine MHV68.ΔHS replication, reactivation, and latency establishment in specific cell types relevant to *in vivo* infection.

The second major defect observed upon MHV68.ΔHS infection is a marked reduction in the number of infected splenocytes present during peak latency establishment relative to mice infected with wild-type or mutant rescue viruses. This defect could arise simply as a downstream consequence of the aforementioned impairment in trafficking. However, significantly reduced levels of viral DNA are detected in the spleen even after an intraperitoneal infection, which bypasses the need for acute replication in the lung and subsequent trafficking through the lymph nodes [37]. Thus, MHV68.ΔHS appears defective in both trafficking and latency establishment in the spleen. Such a phenotype might occur if MHV68.ΔHS-infected cells preferentially entered the lytic cycle, perhaps enabling more efficient clearance by the immune system. This model is supported by our observation that an increased percentage of cultured 3T3 cells infected with MHV68.ΔHS express lytic markers relative to those infected with MHV68.WT. However, if this represents enhanced entry into the lytic cycle, there must be a downstream defect that tempers subsequent viral output, as the host shutoff mutant virus does not replicate to higher titers in 3T3 cells than the wild-type virus. In this regard, host shutoff could be important for optimizing the balance of host or viral proteins in an infected cell, as has been reported in an EBV mutant lacking BGLF5, where altered levels of proteins inhibited viral maturation and egress [64]. One mechanism that could shift the balance of infection away from latency is if muSOX down-regulates factors that inhibit latency establishment; such negative regulators would then accumulate in the presence of the R443I mutant, driving cells into lytic replication. MuSOX activity may also be required for the virus to achieve the appropriate level of host and or viral factors packaged into the viral particle in order to efficiently establish latency in newly infected cells rather than enter the lytic cycle.

The large plaque phenotype observed with MHV68.ΔHS is similar to what is observed following infection with MHV68 constitutively expressing (or over-expressing) the lytic transcription factor RTA [65]. Also similar to MHV68.ΔHS, these viruses replicate to near wild-type levels in the lung but establish latency at lower levels in the spleen. The constitutive RTA-expressing viruses have been found to efficiently traffic to the spleen but fail to be maintained at this site [66]. This attenuation has been ascribed to the viruses producing excessive amounts of the RTA-inducible lytic genes, creating an intracellular environment favoring immediate entry into the lytic cycle rather than entering latency. Thus, during an infection, an important role for muSOX may be to ensure that factors driving lytic replication are not overrepresented. While it is tempting to speculate a connection between the increased percentage of lytically infected 3T3 cells and the latency establishment defect *in vivo* with MHV68.ΔHS, at present the precise relationship between these observations remains unclear.

Given the broad effects of host shutoff, the mechanism of MHV68.ΔHS attenuation is anticipated to deviate from the gene-for-gene interactions associated with many virulence factors. Further research in this area will uncover the likely means by which host shutoff influences latency establishment, as well as reveal novel connections between the lytic and latent stages of the gammaherpesviruses lifecycle.

## Materials and Methods

### PCR Mutagenesis

A hemagglutinin (HA) tag was introduced at the 5′ end of muSOX by PCR using the primers 5′-CGGAATTCATGGCTTACCCATACGATGTACCTGACTATGCGATGGAAG GGTCGATTATTC-3′ and 5′-ATAGTTTAGCGGCCGCTTAGGGGGTTATGGGTTTTCT-3′. HA-muSOX was then cloned into the EcoRI/NotI sites of pCDEF3 containing a T7 promoter to generate pCDEF3-T7-HA-muSOX. MuSOX was randomly mutagenized by PCR with the Genemorph kit (Strategene) according to the manufacturer's protocol using 35 ng of pCDEF3-T7-HA-muSOX as template, the above primers, and 30 PCR cycles to generate a pool of random mutants. The mutants were then cloned into the EcoRI/NotI sites of pCDEF3-T7. R443I was generated by QuickChange (Stratagene) using the primers 5′-GCTCATCATCACTCCTGT TATAATTCCATCTACTGTGCTGC-3′ and 5′-GCAGCACAGTAGATGGAATTATAACAG GAGTGATGATGAGC-3′.

### Cells, Transfections, and Viruses

HEK293T, COS7, NIH 3T3, NIH 3T12, and Vero cells were maintained in Dulbecco's modified Eagle's medium (DMEM; Invitrogen) supplemented with 10% fetal bovine serum (FBS, Invitrogen). 293T cells were transfected with Effectene (Qiagen) following the manufacturer's protocol.

The green fluorescent protein (GFP)-expressing MHV68 bacterial artificial chromosome (BAC) infectious clone has been described elsewhere (RγHV68A98.01 [28]), and mutants were generated by allelic exchange as previously described [67]. To generate the MHV68.ΔHS BAC, a targeting region consisting of 566 nt upstream and 568 nt downstream of the mutation site was ligated into pGS284 between BglII and NotI restriction sites and electroporated into the S17λpir strain of *E. coli*. The MHV68-YFP BAC infectious clone was a generous gift from Dr. Samuel Speck (Emory University) [29], and MHV68-YFP.ΔHS was generated using the same methodology as MHV68.ΔHS. The targeting vector for the mutant rescue BAC (MHV68.MR) was generated by ligating the region around wild-type muSOX into pGS284. Targeting vector-containing cells were cross-streaked with BAC-containing GS500 cells and successful recombinants were identified by colony PCR and subsequent digest with PsiI. BAC DNA was isolated from positive clones using the Qiagen Large-Construct kit (Qiagen). BAC variants were verified by restriction digests with EcoRI and PsiI and sequencing of the region surrounding the recombination site. BAC-derived MHV68 virus was produced by transfecting 2 µg of BAC DNA into NIH 3T3 cells using SuperFect (Qiagen). Virus was then amplified in NIH 3T12 cells and titered by plaque assays on NIH 3T3 cells. Before infecting mice, the loxP-flanked BAC vector sequence was removed from the recombinant viruses by passaging the virus over Vero cells expressing Cre recombinase (kindly provided by Dr. Samuel Speck, Emory University) [59], and BAC removal was confirmed by PCR analysis.

### Immunofluorescence Assays

MuSOX expression and PABPC or HA-muSOX localization were analyzed as described previously [16], [23]. Briefly, NIH 3T3, HEK293T, or COS7 cells were grown on coverslips, and infected with MHV68 variants or transfected with HA-muSOX variants. Cells were then stained with rabbit polyclonal anti-muSOX (1:25 dilution), mouse monoclonal anti-PABPC 10e10 (1∶25 dilution) (Santa Cruz Biotechnology), or mouse monoclonal anti-HA (1:500 dilution) (Abcam) and AlexaFluor 546- or 488-conjugated goat anti-mouse or goat anti-rabbit secondary antibody (1∶1500). Coverslips were mounted in DAPI-containing Vectashield mounting medium (Vector Labs) to stain cell nuclei.

For flow cytometry analysis, NIH 3T3 cells were infected with the indicated virus and, 18 hpi, cells were washed with PBS and harvested via trypsin digestion. Cells were fixed in a 4% formaldehyde solution, permeabilized in 1% Triton X-100 and 0.1% sodium citrate in PBS, and incubated with anti-muSOX antibodies at a 1:12.5 dilution in 1% Triton X-100, 0.5% Tween, and 3% BSA in PBS. Stained cells were then washed in PBS and incubated with goat anti-rabbit antibodies conjugated to PE-Cy5.5 (Invitrogen) at a dilution of 1:150. Data were collected on an EPICS XL cytometer (Beckman-Coulter) and analyzed using FlowJo software (Tree Star).

### Western Blots and Northern Blots

For Western blotting, cell lysates were prepared in RIPA buffer [50 mM Tris-HCl, pH 7.4, 150 mM NaCl, 2 mM EDTA, 1% Nonidet P-40 (vol/vol), 0.1% SDS (w/vol)] containing Protease Inhibitor Cocktail (Roche) and quantified by Bradford assay (Bio-Rad). Equivalent amounts of each sample were resolved by SDS-PAGE, transferred to a PVDF membrane, and Western blotted with anti-HA 12CA5 monoclonal antibodies (Invitrogen; 1∶5,000) and HRP-conjugated goat anti-mouse secondary antibodies (Southern Biotechnology; 1∶5,000).

For Northern blotting, total RNA was harvested using RNA-BEE (Tel-Test) and resolved by agarose-formaldehyde gel electrophoresis. RNAs were transferred to a 0.45 µm nylon membrane and probed with ^32^P-labeled GFP DNA probes generated using the Rediprime II random prime labeling system (GE Healthcare).

### DNase Assays

DNase activity of the muSOX variants was performed as described previously [16]. Briefly, muSOX variants were *in vitro* transcribed with the mMessage mMachine T7 Kit (Ambion) and translated using rabbit reticulocyte lysates (Promega). Translational product was incubated with linear DNA at 37°C for the indicated time period and then the DNA was phenol/chloroform extracted, and resolved by agarose gel electrophoresis. One-sixth each IVT reaction was also separated by SDS-PAGE. The gels were then fixed, dried, and visualized by autoradiography to verify equivalent protein expression.

### Quantitative PCR

To quantify RNA, we isolated RNA from transfected cells using RNA-Bee (Tel-Test) or the Zymo Mini RNA II Isolation Kit (Zymo Research). Samples were treated with Turbo DNase (Ambion) according to the manufacturer's protocol to remove genomic DNA contamination. To generate cDNA, RNA samples were reverse transcribed using AMV RT (Promega) and an oligo dT or 18S-specific primer. 18S rRNA or GAPDH mRNA levels were quantified using TaqMan ribosomal RNA control reagents or TaqMan rodent GAPDH control reagents (Applied Biosystems). Other endogenous mRNA levels were quantified using Applied Biosystems Taqman Gene Expression Assays (actB-Mm01205647_g1, rplp2-Mm03059047_gH, tubb5-Mm00495806_g1). To quantify viral genomes, DNA was first isolated from spleen cells or cervical lymph node cells by the Qiagen QIAamp DNA Mini Kit following the manufacturer's protocol. Viral DNA levels were then measured using a previously described assay targeting the MHV68 ORF8 gene [35]. All qPCR reactions were performed with TaqMan Universal PCR Master Mix.

### Cell Death Assays

To measure levels of lactate dehydrogenase (LDH) released into the media during an infection, NIH 3T3 cells were infected at an MOI of 5 for 16.5 h. Infection supernatant (60 µl) was mixed with 60 µl LDH detection reagent in triplicate in a 96-well plate as described previously [68]. Absorbance was read on an Elisa-reader at 490 nm wavelength. To detect caspase activity, NIH 3T3 cells were infected at an MOI of 5 for 24 h, whereupon caspase activity was measured using the Caspase-Glo 3/7 Assay System (Promega) following the manufacturer's protocol. To monitor Annexin V-PE and 7-AAD staining by flow cytometry, NIH 3T3 cells were infected at an MOI of 10 for 18 h. Cells were then washed with PBS and harvested via trypsin digestion. Infection supernatant, PBS wash, and cells were combined and pelleted by centrifugation. Cells were washed in PBS and then stained for Annexin V-PE (BD Biosciences, Material # 556422) and 7-AAD (BD Biosciences, Material # 559925) via the manufacturer's protocol using 3 µl of each dye. Data were collected on an EPICS XL cytometer (Beckman-Coulter) and analyzed using FlowJo software (Tree Star).

### *In vivo* Infections and Experiments

Female C57BL/6J mice were obtained from The Jackson Laboratory (Bar Harbor, ME) and infected when 4–6 weeks old. Mice were anesthetized with isoflourane and inoculated intranasally with 5×10^4^ plaque forming units (pfu) in 20 µl DMEM (Invitrogen). For intraperitoneal infections, 1×10^3^ pfu in 0.2 ml PBS were injected into the peritoneal cavity of mice. Lungs were harvested at 3, 5, or 7 dpi, homogenized with a tissue homogenizer for 1 minute at 24,000 rpm in 10 ml DMEM with 10% FBS, 100 U of penicillin per ml, and 100 mg of streptomycin per ml (Pen/Strep, Invitrogen). Spleens were harvested at 17 or 19 dpi. To identify any preformed infectious particles, half of each spleen was homogenized as above in 5 ml DMEM with 10% FBS, and Pen/Strep. The tissue homogenate was then assayed for viral particles by plaque assay on monolayers of NIH 3T3 cells overlaid with 1% agarose for 4 days. The cells were then fixed and stained with 0.04% methylene blue. Splenocytes were isolated from the other half of the spleen by dissociating the spleen and passing through a 40 µm cell strainer (BD-Falcon). Cells were then pelleted, resuspended in red blood cell lysis buffer (150 mM NH_4_Cl, 10 mM KHCO_3_, 0.1 mM EDTA), and incubated at room temperature for 5 minutes. Cells were again pelleted and resuspended in RPMI medium with 10%FBS and Pen/Strep before counting. Cervical lymph nodes were harvested at 10 dpi and isolated in the same manner as splenocytes, but without lysing the red blood cells.

### Infectious Center Assay

The number of reactivating splenocytes was determined as described previously [34]. Briefly, 21,000 NIH 3T3 cells were plated per well in a 24-well plate the day before infection. 2.5×10^6^, 5×10^5^, or 1×10^5^ splenocytes were overlayed onto the NIH 3T3 cells and incubated for 4 days. Cells were then fixed with 10% formaldehyde and stained with 0.04% methylene blue to visualize plaques.

### Limiting Dilution PCR

The frequency with which cells latently harbor the viral genome was determined as described previously [36]. Briefly, 4-fold serial dilutions of splenocytes were plated in 96-well plates with 16 wells per dilution. Nested PCR for the ORF50 gene was performed and the resulting PCR product was run on an agarose gel. Wells positive for a PCR product by ethidium bromide staining contain at least one copy of the viral genome. Single-copy sensitivity was confirmed using serial dilutions of MHV68 BAC DNA.

### Graph Design and Statistical Analysis

All graphs were designed and statistical analysis performed using the GraphPad Prism Software version 4 or 5. Data were analyzed for statistical significance using the Student's t-test or, for *in vivo* data, the Mann-Whitney nonparametric test. For limiting dilution PCR analysis, data were subject to nonlinear regression using the log(agonist) vs. response curve with a nonvariable slope.

### Ethics Statement

This study was carried out in strict accordance with the recommendations in the Guide for the Care and Use of Laboratory Animals of the National Institutes of Health. The protocol was approved by the Committee on the Ethics of Animal Experiments of the University of California Berkeley (Permit Number: R292-0507). All animals were anesthetized prior to infection with isoflurane, and all efforts were made to minimize suffering.

## Supporting Information

Figure S1 **Levels of muSOX expressing cells increase upon MHV68.ΔHS infections.** 3T3 cells infected at an MOI of 1 with either MHV68-YFP or MHV68-YFP.ΔHS were analyzed at 18 hpi for YFP and muSOX expression by flow cytometry using anti-muSOX antibodies. The percentage of cells within each quadrant is indicated. Shown is a representative graph of four independent experiments.
(EPS)

Figure S2 **ORF54 transcript accumulates to higher levels during a MHV68.ΔHS infection.** 3T3 cells were infected at an MOI of 5 with MHV68.WT, MHV68.ΔHS, or MHV68.MR. At the indicated times post infection total RNA was isolated and levels of ORF54 mRNA and 18S rRNA were quantified by RT-qPCR. Shown are the ORF54/18S ratios normalized to wild-type infection at 18 hpi along with the mean and standard deviation from at least five independent experiments. The p-values comparing MHV68.ΔHS to MHV68.MR are indicated. “n.s.” indicates that the p-value is greater than 0.05.
(EPS)

Figure S3 **MHV68.ΔHS does not induce cell death.** (A) 3T3 cells were infected for 16.5 h with MHV68.WT, MHV68.ΔHS, or MHV68.MR at an MOI of 5, whereupon levels of lactate dehydrogenase released into the media were quantified by an enzymatic color change assay. Triton X-100 was added at 1% to lyse cells as a positive control. Shown are the mean and standard deviation from three experiments. (B) 3T3 cells were infected as described above but for 24 h, then levels of caspase3/7 activity were quantified via a luciferase-based luminescent assay. Etoposide (ETP) was included at 25 µM as a positive control. Shown is the luciferase signal strength normalized to the mock infected control along with the mean and standard deviation from three independent experiments. The p-value comparing MHV68.ΔHS to MHV68.MR is indicated. “n.s.” indicates that the p-value is greater than 0.05. (C) 3T3 cells were infected at an MOI of 10 and analyzed for Annexin V-PE and 7-AAD staining 18 hpi by flow cytometry. ETP was included at 25 µM as a positive control. The percentage of cells within each quadrant is indicated.
(EPS)

Table S1 **Amino acid mutations introduced into muSOX.**
(PDF)
