# Supplementary material for: Global mRNA Degradation during Lytic Gammaherpesvirus Infection Contributes to Establishment of Viral Latency
Source: PLoS Pathog. 2011 Jul 21;7(7):e1002150. doi: 10.1371/journal.ppat.1002150 (PMC3141057; doi:10.1371/journal.ppat.1002150)
Supplement: Table S1 — Amino acid mutations introduced into muSOX. (PDF) [file ppat.1002150.s004.pdf]

**Supplemental Table. Phenotype of muSOX mutants.**

| | Expression | Host Shutoff | DNase | Source |
|---|---|---|---|---|
| P174S | + | + | N.D. | SOX P176S Homolog [25] |
| V185A | + | + | N.D. | EBV V169A Homolog [24] |
| Q127H | + | + | - | SOX Q129H Homolog [25] |
| R62G | - | - | N.D. | Conserved amongst γ-HV[a] |
| F160V | - | - | N.D. | Conserved amongst γ-HV |
| H169L | + | + | N.D. | Conserved amongst γ-HV |
| R409S | + | - | - | Conserved amongst γ-HV |
| Δ314-318 | + | - | - | Putative NLS[b] |
| R443I | + | - | + | Screen |

N.D. = Not Determined

a. γ-HV = gammaherpesviruses

b. NLS = Nuclear Localization Signal
